# Changing phenotype, early clinical course and clinical predictors of inflammatory bowel disease in Sri Lanka: a retrospective, tertiary care-based, multi-centre study

**DOI:** 10.1186/s12876-021-01644-5

**Published:** 2021-02-16

**Authors:** M. A. Niriella, I. K. Liyanage, S. K. Kodisinghe, A. P. De Silva, A. V. G. A. M. Jayatissa, N. M. M. Navarathne, R. K. Peiris, U. P. Kalubovila, S. R. Kumarasena, R. W. Jayasekara, H. J. de Silva

**Affiliations:** 1 Department of Clinical Medicine, Faculty of Medicine, University of Kelaniya, Ragama, Sri Lanka; 2 Faculty of Medical Sciences, University of Sri Jayewardenepura, Nugegoda, Sri Lanka; 3 University Medical Unit, Colombo North Teaching Hospital, Ragama, Sri Lanka; 4 Gastroenterology Unit, National Hospital of Sri Lanka, Colombo, Sri Lanka; 5 Gastroenterology Unit, Colombo South Teaching Hospital, Kalubovila, Sri Lanka; 6 Gastroenterology Unit, Teaching Hospital, Kandy, Sri Lanka; 7 Gastroenterology Unit, Teaching Hospital Karapitiya, Galle, Sri Lanka; 8 Human Genetics Unit, Faculty of Medicine, University of Colombo, Colombo, Sri Lanka

**Keywords:** Inflammatory bowel disease, Ulcerative colitis, Crohn disease, Phenotype, Clinical course, Clinical predictors, Sri Lanka

## Abstract

**Background:**

Inflammatory bowel disease (IBD) is increasing in the Asia-Pacific region, with changes in disease phenotype and course. We aimed to assess the changing phenotypes of IBD over ten years, describe the early clinical course (ECC) and identify the clinical predictors (CP) of poor outcomes among a large, multi-centre, cohort of Sri Lankan IBD patients.

**Methods:**

We included patients [diagnosed between June/2003–December/2009-Group-1(G1), January/2010–June/2016-Group-2(G2)] with ulcerative colitis (UC) and Crohn disease (CD) from five national-referral centres. Changing phenotype from G1 to G2, ECC (disease duration < 3-years) and CP of poor outcomes (disease duration ≥ 1-year) was assessed. Poor outcomes were complicated-disease (CompD-stricturing/penetrating-CD, extensive-UC/pancolitis, perforation/bleeding/colectomy/malignancy) and treatment-refractory disease (TRD-frequently-relapsing, steroid-dependent/refractory and biologic use).

**Results:**

375 (UC-227, CD-148) patients were recruited. Both G1/G2 had more UC than CD (77% vs 23%, 54.5 vs 45.5 respectively, *p* < 0.01). Increase of CD from G1-to-G2 was significant (23–45.4%, *p* < 0.001). In both groups, left-sided colitis (E2) and ileo-colonic (L3)/non-stricturing, non-penetrating disease behaviour (B1) CD predominated. Extensive-colitis (E3) (36.4% vs 22.7, *p* < 0.05) and stricturing-CD (B2) (26.1% vs 4.0%, *p* < 0.01) was commoner in G1. ECC was assessed in 173-patients (UC-94, CD-79). Aggressive disease behaviour and TRD were low among both UC and CD. Immunomodulator use was significantly higher among CD than UC (61.5% vs 29.0% respectively, *p* < 0.01). Anti-TNF use was low among both groups (UC-3.2%, CD-7.7%). Disease complications among UC [bleeding (2.1%), malignancy-(1.1%), surgery-(2.1%)] and CD [stricture-(3.9%), perforation-(1.3%), malignancy-(1.3%), surgery-(8.9%)] were generally low. CPs were assessed in 271-patients (UC-163, CD-108). Having a family history of IBD (for UC), extraintestinal manifestation (EIM), severe disease at presentation, being in younger age categories and severe disease at presentation, (for both UC and CD) predicted poor outcomes.

**Conclusion:**

There was an increase in CD over time without change in disease phenotype for both UC and CD. A relatively benign ECC was observed. Family history (UC), EIMs (UC/CD), severe disease at presentation (UC/CD), younger age (CD/UC) CPs of poor outcomes.

## Background

Inflammatory bowel disease (IBD), comprising ulcerative colitis (UC) and Crohn disease (CD), are chronic inflammatory conditions of the gastrointestinal tract imposing a significant disease burden worldwide. Pathogenesis of IBD is multifactorial where genetic predisposition, gut microbiota, and other environmental factors contribute [1]. This resulted in distinct epidemiological trends over time and across geographical regions. IBD was initially considered a disease of wealthy areas including Europe, North America and Australasia [2]. Initially, UC was commoner in most of these countries. Later CD gradually increased in incidence and in some parts of the world it has surpassed UC in rate [3]. IBD is increasingly detected in the emerging economies of the world, such as in China, Singapore, South Korea and India [1]. Although data on the evolution of epidemiological and morphological characteristics of IBD is available from some of these countries, a comparative paucity of evidence is noted from the South-Asian region.

In Europe, the overall annual incidence of IBD per 100,000 population is reported to be 3–7 cases for CD and 4–11 cases for UC [4]. The Asia Pacific Crohn’s and Colitis Epidemiology Study (ACCESS) carried out in Australia, China, Hong Kong, Indonesia, Macau, Malaysia, Singapore, Sri Lanka, and Thailand showed a rapid rise in the incidence of IBD [5]. The crude annual incidence per 100,000 is estimated to be 0.76 for UC and 0.54 for CD in the ACCESS study [6, 7]. Considering the scarcity of more representative studies, there is a need for understanding the transition of phenotypes and disease characteristics from the early course of the disease epidemiology from low- and middle-income countries.

Sri Lanka does not have a National IBD registry, and there are only a few tertiary referral centres carting to specialist care of IBD patients. Therefore, a study involving all the major referral centres in the country would be best reflective of a nationwide analysis of IBD and its care in Sri Lanka in the absence of a national registry. The objectives of the present study were to assess the changing phenotypes of IBD over ten years, describe the early clinical course (ECC) of IBD and identify the clinical predictors (CP) of poor outcomes among a large, multi-centre, cohort of Sri Lankan IBD patients.

## Methods

We conducted a cross-sectional, multicenter-study among adult patients (> 16 years of age) with histologically confirmed IBD from five national referral centres in three major cities in Sri Lanka. We recruited patients from Gastroenterology Units of Colombo North Teaching Hospital, Ragama, National Hospital of Sri Lanka, Colombo, Colombo South Teaching Hospital, Kalubovila, Teaching Hospital, Kandy and Teaching Hospital Karapitiya, Galle, Sri Lanka. These centres collectively provide tertiary level specialist Gastroenterology care for the majority of Sri Lankan IBD patients. The study was conducted from June 2014 to June 2016. From each centre, we recruited all consecutive, consenting patients with IBD during the period of the study.

The patients were diagnosed with UC or CD based on clinical, endoscopic and histological features. We excluded patients with other inflammatory or infective bowel conditions such as infective colitis, indeterminate colitis or intestinal TB.

Data were obtained using an interviewer-administered, structured questionnaire. In addition, clinical data were collected by review of medical records. Phenotypic data (type, location, severity, treatment types, response to treatment and complications) of patients were recorded. Comorbid conditions, details on disease and treatment were confirmed using medical records whenever possible. Montreal classification of IBD was used for the study [8]. Therefore, severe disease at presentation was defined as E3 or S3 for UC and multi-segmental disease, B2 or B3 or presence of P modifier for CD.

We defined complicated disease (CompD) as having stricturing or penetrating disease among patients with CD and extensive or pancolitis in patients with UC. Perforation, significant bleeding, requirement of colectomy or malignant change having taken place were also considered as CompD.

Patients with a disease course that was frequently relapsing (two or more episodes of worsening symptoms after a period of remission), steroid-dependent (who fails to taper steroids below 10 mg within 16 weeks from a starting dose of 0.75–1 mg/kg oral prednisone-equivalent, or who relapses within 12 weeks after steroid discontinuation), steroid-refractory (a patient not responding to 0.75–1 mg/kg of oral prednisone-equivalent within two to four weeks) or requiring biologics were classified as a treatment-refractory disease (TRD) [9].

We grouped patients according to the time of diagnosis as follows: Group-1(G1)-diagnosed between June/2003–December/2009, Group-2(G2)-January/2010–June/2016. We considered patients with a disease duration of less than three years to be in the ECC. We analyzed CP of CompD and TRD among those with one year or more follow up period.

Data were analyzed with STATA version 13. Suitable parametric and non-parametric tests were used, and a *p *value of < 0.05 was considered statistically significant. Odds ratios and 95% confidence intervals were obtained by univariate logistic regression analysis.

Ethical approval for the study was obtained from the Ethical Review Committee (ERC) of the Faculty of Medicine, University of Kelaniya, Ragama, Sri Lanka and individual hospital ERCs.

## Results

A total of 375 patients (UC-227, CD-148) were included in the study. Six patients with indeterminate colitis (IBD unclassified) were excluded from the analysis. Mean age was 44.9 (SD = 15.9) years, and 50.1% were males. These patients were diagnosed between 2003 and 2016. Characteristics of the patients diagnosed in the period between June-2003–December-2009 (group 1) and January-2010 to June-2016 (group 2) are shown in Table 1. The average age at diagnosis was 38.6 (SD = 0.7) years (UC = 40.3 years CD = 35.4 years, *p* < 0.01). 100 (26.7%) patients belonged to G1, and 275 (73.3%) patients belonged to G2. Both G1 and G2 had a higher proportion of UC compared to CD (77% vs 23%, *p* < 0.01 and 54.5 vs 45.5, *p* < 0.01 respectively).

**Table 1:** Demographic and disease characteristics of patients diagnosed between June 2003 and December 2009 (Group 1) and January 2010–June 2016 (Group 2)

Characteristic	Group 1 (n = 100)	Group 2 (n = 275)	*p* value
*Overall *(*n = 375*)			
Male gender	50 (50.0)	138 (51.2)	0.83
Age at diagnosis (mean, SD)	46.0 (14.5)	43.6 (16.6)	0.19
Ulcerative colitis	77 (77.0)	150 (54.5)	** < 0.01**
Crohn's disease	23 (23.0)	125 (45.5)	** < 0.01**
*Ulcerative colitis *(*n = 227*)			
Complicated disease (CompD)	29 (36.2)	40 (32.0)	0.14
Treatment refractory disease (TRD)	4 (5.2)	17 (13.6)	0.05
Disease extent—proctitis	11 (14.3)	29 (19.3)	0.35
Disease extent—left sided colitis	38 (49.4)	87 (58.0)	0.22
Disease extent—extensive colitis	28 (36.4)	34 (22.7)	**0.02**
*Crohn’s disease *(*n = 148*)			
Complicated disease (CompD)	14 (60.8)	34 (27.6)	0.07
Treatment refractory disease (TRD)	4 (17.4)	20 (16.4)	0.91
Disease extent—confined to ileum	4 (17.4)	29 (24.6)	0.46
Disease extent—confined to colon	3 (13.0)	32 (27.1)	0.15
Disease extent—ileocolonic disease	15 (65.2)	57 (48.3)	0.14
Perianal disease	7 (30.4)	28 (22.8)	0.43
Disease behaviour—inflammatory	16 (69.5)	105 (84.0)	0.10
Disease behaviour—stricturing	6 (26.1)	5 (4.0)	** < 0.01**
Disease behaviour—fistulating	1 (4.3)	15 (12.0)	0.28

Values are reported as number (percentages). *p* values obtained using Chi2 test for binary variables

Bold values indicate significant at *p* < 0.05

The increase of proportion of CD from G1 to G2 was significant (23% to 45.4%, *p* < 0.001). In both groups (G1 and G2), left-sided colitis (E2) (49.4% and 58.0%) for UC and ileo-colonic (L3) (65.2% and 48.3%)/non-stricturing, non-penetrating disease behavior (B1) (69.5% and 84%) for CD predominated. Extensive colitis (E3) for UC (36.4% vs 22.7, *p* < 0.05) and stricturing-CD (B2) (26.1% vs 4.0%, *p* < 0.01) was commoner in G1.

A total of 173 patients (UC-94, CD-79) were within three years of initial diagnosis and was used for assessing the ECC. In this group, there were 94 (54.3%) patients with UC and 79 (45.7%) patients with CD. Disease characteristics of this group of patients are described in Table 2. Age (SD) at diagnosis was higher for UC compared to CD (42.7 vs 34.2 years respectively, *p* < 0.05).

**Table 2:** Characteristics of the patients in early clinical course (ECC) of UC and CD (within 3 years of diagnosis)

Characteristic	UC (n = 94)	CD (n = 79)	P value
*Gender*			
Male	49	43	0.76
Female	45	36	
Age at diagnosis in years [median and IQR]*	42.7 (29.3–55.9)	34.2 (22.4 – 51.3)	0.04
Duration of diagnosis in months [median and IQR]*	21 (14–27)	21 (14–28)	0.99
Duration of follow up in months [median and IQR]*	18 (5–31)	9 (2–27)	0.01
*Disease behavior *[*number *(*%*)]			
Admission with severe episode	20 (21.3)	9 (11.4)	0.28
Extensive (E2) or Pancolitis (E3)	26 (27.7)	–	–
Stricturing (B2) or penetrating (B3) disease	–	9 (11.4)	–
Perianal disease (P)		16 (20.5)	–
*Treatment refractoriness *[*number *(*%*)]			
Steroid dependent	1 (1.1)	0	0.36
Steroid refractory	0	1 (1.3)	0.27
Frequently relapsing	9 (9.6)	6 (7.6)	0.64
*Highest therapy*			
Immunomodulators	27 (29.0)	48 (61.5)	< 0.01
Anti-TNF agents	3 (3.2)	6 (7.7)	0.19
*Disease complications*			
Stricture		3 (3.9)	
Perforation		1 (1.3)	
Bleeding	2 (2.1)	0	
Surgery	2 (2.1)	7 (8.9)	
Malignancy	1 (1.1)	1 (1.3)	

Immunomodulator use was significantly higher among CD compared to UC (61.5% vs 29.0% respectively, *p* < 0.01) and anti-TNF use were low among both groups (3.2% and 7.7% respectively for UC and CD). Complications among UC [bleeding (2.1%), malignancy-(1.1%), surgery-(2.1%)] and among CD [stricture-(3.9%), perforation-(1.3%), malignancy-(1.3%), surgery-(8.9%)] were generally low during the ECC.

CPs for poor outcomes were assessed in 271 (UC-163, CD 108). Results of binary logistic regression analyses in the patients with UC and CD on associations of CompD, and TRD are summarized in Tables 3 and 4. Having a family history of IBD (OR 3.35), extraintestinal manifestation (OR 1.76) and severe disease at presentation (OR 2.22) were associated with poor outcomes in UC. Patients in higher age categories (> 50 years) was less likely to have complicated disease and patients above the age of 30 years were less likely to have TRD in UC. Having extraintestinal manifestations and severe disease at presentation predicted poor outcomes in CD.

**Table 3:** Predictors of poor outcomes in UC

Characteristic	Complicated disease	Treatment refractory disease
Male gender	1.23 (0.77–1.95)	0.88 (0.43–1.78)
Age < 30 years	–	
30–50 years	0.62 (0.32–1.22)	0.99 (0.41–2.42)
> 50 years	**0.48 (0.24–0.96)**	0.69 (0.26–1.79)
Having tertiary education	**1.71 (0.93–3.14)**	1.22 (0.51–2.91)
Employed	1.77 (0.73–1.89)	1.34 (0.66–2.74)
Ever smoker	0.90 (0.44–1.86)	2.11 (0.83–5.37)
Appendicectomy	0 .57 (0.06–5.21)	1.98 (0.43–9.06)
Family history of IBD	**3.35 (1.32–8.47)**	1.16 (0.26–5.14)
Diagnosed after 2009	0.76 (0.47–1.21)	1.54 (0.68–3.49)
Diabetes	0.66 (0.33–1.34)	9 .99(0.34–2.92)
Obesity (BMI > 25)	0.86 (0.52–1.42)	1.8 (0.89–3.69)
Extra intestinal	**1.76 (1.09–2.83**)	**2.20 (1.08–4.48)**
Severe disease at presentation	**2.22 (1.17–4.20)**	2.25(0.97–5.22)

Expressed as odds ratio (OR) and standard deviation (SD)

Bold values indicate significant at *p* < 0.05

**Table 4:** Predictors of complicated disease, disease with complications and treatment refractory disease in CD

Characteristic	Complicated disease	Treatment refractory disease
Male gender	1.45 (0.78–2.70)	1.63 (0 .72–3.69)
Age < 30 years		
30–50 years	1.35 (0.66–2.76)	**0.16 (0.062–0.43)**
> 50 years	0.47 (0.20–1.11)	**0.06 (0.01–0.25)**
Having tertiary education	0.81 (0.39–1.71)	1.13 (0.41–3.08)
Employed	1.24 (0.65–2.37)	0.68 (0.28–1.67)
Ever smoker	1.39 (0.42–4.58)	0.77 (0.18–3.34)
Appendicectomy	1.38 (0.42–4.52)	–
Family history of IBD	1.44 (0.31–6.63)	1.60 (0.36–7.15)
Diagnosed after 2009	0.28 (0.13–0.62)	2.62 (0.88–7.75)
Diabetes	0.14 (0.02–1.13)	–
Obesity (BMI > 25)	1.02 (0.50–2.11)	**0.19 (0.05–0.83)**
Extra intestinal involvement	**2.68 (1.42–5.09)**	1.17 (0.51–2.66)
Severe disease at presentation	**25.78 (5.75–115.49)**	**3.37 (1.40–8.15)**

Expressed as odds ratio (OR) and standard deviation (SD)

Bold values indicate significant at *p* < 0.05

## Discussion

In the present study, there was an increase in CD over time, but no changes in disease phenotype for either UC or CD. Left-sided UC and ileocolonic CD were the main phenotypes. Only a few patients had CompD or TRD for both UC and CD indicating a relatively benign ECC within three years of diagnosis. Family history of IBD, younger age, presence of EIMs and severe disease at presentation were the CPs of poor outcomes in this cohort.

Our findings showed a near doubling of the proportion of patients with CD (from 23 to 46%) from 2003/2009 to 2010/2016. Similar epidemiological transitions were observed in Western countries during the early phase of the epidemic of IBD [2] and in some Asian countries where IBD is recently rising in prevalence [10, 11]. Although an exact mechanism for the proportional increase in CD compared to UC over time in a given population is not well understood, a multitude of environmental changes, dietary patterns along with genetic and epigenetic factors may contribute [2]. Further, CD was also the diagnosis in 45.7% (79/173) of the patients who were in the ECC. This percentage of CD in G2 and ECC is significantly a higher proportion compared to the previously reported value of 18.6% CD (55/295) from Sri Lanka [12] but is comparable to the figures of 40.1% (1606/4006) reported in a recent study from India [13].

Scientists first noted changes in disease patterns of IBD along with the potential role of environmental and genetic interactions more than fifteen years ago when they observed second-generation South Asian migrants having an equal risk of UC as their western counterparts [14]. Another study in a pediatric population in British Columbia demonstrated a higher prevalence and different phenotypic characteristics including more extensive colonic disease and male predominance in South Asian migrant populations compared to Caucasians [15].

In Caucasian patients, the average age of diagnosis of CD had been between 33 and 45 years while the average age of diagnosis of UC was approximately five years later [16, 17]. Our findings agreed to the same and the mean age at diagnosis was lower for CD compared to UC (34.2 and 42.7 years respectively). We observed a lower mean age at diagnosis for G2 compared to G1 (43.6 and 46.0 years, respectively). This finding is similar to the evidence from the Western world where more and more younger people are detected to have IBD, especially UC [18, 19].

In both G1 and G2, left-sided colitis/proctosigmoiditis (E2) for UC (49.4% and 58.0% respectively) and inflammatory phenotype (B1) (69.5% and 84.0% respectively) and ileocolic location (L3) (65.2% and 48.3% respectively) for CD predominated indicating no change in disease phenotype over time. Comparable disease phenotype predominance for both UC (51% for E2) and CD (67.8% B1 and 40.9% L3) has been reported recently from India [13].

Patients diagnosed after 2009 were less likely to have extensive colitis for UC and less likely to have structuring disease behaviour for CD. Shorter duration of disease, earlier detection and availability of better treatment may have contributed to this pattern.

Patients with CD in their ECC showed less aggressive disease behaviour compared to Caucasian patients. They had a lesser prevalence of stricturing, penetrating and fistulating CD behaviour [16, 20]. Among patient with an early course of UC, the prevalence of pan-colitis was similar to Caucasian populations at approximately one-fifth of all patients [21, 22]. Patients in their ECC had low (< 30%) CompD, low (< 10%) TRD indicating a relatively benign ECC. Previous evidence from Sri Lanka also points towards milder disease in both UC and CD in local patients compared to western populations [10]. In a hospital-based survey in Sri Lanka, 26.2% of patients with UC had extensive large bowel involvement, and 9% had a severe disease at presentation. Out of patients with CD, perianal disease and upper gastrointestinal involvement was seen in 1.6% each and severe disease was observed in 20.0% [10].

Significantly, a higher proportion of patients with CD were on immunomodulatory therapy compared to UC, during the ECC. A very low percentage (< 8% for CD and < 4% for UC) were on biological therapies in the ECC. These findings again favour a relatively benign ECC for both UC and CD in our cohort.

Identifying characteristics associated with poor outcomes can help to direct treatment in patients with IBD. In this population presence of EIMs and severe disease at presentation were associated with a poorer outcome for both UC and CD. This is in agreement of current understanding of UC and CD in Caucasian populations [21, 22]. We found that most of the other established associations of poor outcomes for IBD were not present in our sample of patients. In Caucasian populations, some predictors of poor outcome in UC were extensive disease at the time of presentation, having extraintestinal manifestations, younger age at diagnosis, the severity of mucosal inflammation and inadequate response to initial treatment [21]. Younger age at presentation, presence of disease complications (stricturing disease, penetrating disease, the involvement of the proximal gastrointestinal tract), need of systemic steroids, tobacco smoking and appendectomy have been noted as indicators of poor outcome in patients with CD [23, 24].

Having a positive family history was associated with the development of CompD in UC this study. However, most of the current evidence suggested that although having a family history increases the likelihood of developing UC but does not influence the prognosis or disease course [25]. In the present study, for patients with CD, a family history of IBD was not associated with having poor outcomes. Numerous authors have observed more severe and complicated CD in patients with a family history of IBD. In a large group of patients with CD followed up for a period of over five years, Laurent Beaugerie reported that younger age (< 40 years) at the onset, disease that involves both small and large intestine, perianal disease and requirement of systemic steroids during the first episode was predictive of disabling disease [23].

There are several limitations to the study. The present study only included tertiary referral care centres, which means that there will be selection bias not reflective of the general population. Milder cases of IBD, either misdiagnosed, presenting to and being managed in the primary or secondary care, as well as milder cases on traditional alternative therapies is not reflected in this study. The data presented are retrospective, so the cumulative probability of an outcomes could not be evaluated. Despite these limitations, this is the largest study to report the IBD demographics and phenotype, ECC and CPs from Sri Lanka [12]. Since we have included the five major tertiary care referral centre in the country with specialist services for IBD, the present study may be considered a nation-wide representative sapmle of symptomatic cases of IBD from Sri Lanka.

The age-adjusted incidence of IBD in Sri Lanka, from a study conducted between April 2011 to March 2012, was 1.55 per 100,000 persons (0.94 for UC and 0.56 for CD) [26]. These values were obtained by a prospective, hospital surveillance study in one district of Sri Lanka. The data we present here are on patients admitted to the main tertiary referral hospitals that treat IBD in Sri Lanka over two periods (June 2003 to December 2009 and January 2010 to June 2016). As such, there is a referral bias and these values cannot be compared to the incidence data.

## Conclusions

Our results confirm the increasing prevalence of CD compared to UC overtime during the early phase of the IBD epidemic. Characteristics of patients during their early stage of the illness and associations of poor outcomes were inconsistent among South Asians compared to Caucasian populations. Severe initial episode and extraintestinal manifestations were associated with poor outcome in both CD and UC. Having a positive family history was associated with CompD in UC but not in CD. The findings may suggest that there is a difference in disease patterns and disease associations between Western or Caucasian populations and South Asians.

## Data Availability

The de-identified datasets used and analyzed during the current study are only available for a valid request to the corresponding author, after notification to and approval of the ERC, Faculty of Medicine, University of Kelaniya, Sri Lanka.
